# Lysozyme–AuNPs Interactions: Determination of Binding Free Energy

**DOI:** 10.3390/nano11082139

**Published:** 2021-08-22

**Authors:** Axel Gomes, Jose M. Carnerero, Aila Jimenez-Ruiz, Elia Grueso, Rosa M. Giráldez-Pérez, Rafael Prado-Gotor

**Affiliations:** 1 Department of Physical Chemistry, Faculty of Chemistry, University of Seville, 41012 Seville, Spain; axelgomes314@hotmail.fr (A.G.); jcarnerero2@us.es (J.M.C.); ajimenez28@us.es (A.J.-R.); elia@us.es (E.G.); 2 Department of Cellular Biology, Physiology and Immunology, Faculty of Science, University of Córdoba, 14014 Cordoba, Spain; rgiraldez@uco.es

**Keywords:** binding free energy, gold nanoparticles, lysozyme, nanoparticle size, surface plasmon resonance

## Abstract

Investigation and optimization of lysozyme (Lys) adsorption onto gold nanoparticles, AuNPs, were carried out. The purpose of this study is to determine the magnitude of the AuNPs–lysozyme interaction in aqueous media by simple spectrophotometric means, and to obtain the free energy of binding of the system for the first time. In order to explore the possibilities of gold nanoparticles for sensing lysozyme in aqueous media, the stability of the samples and the influence of the gold and nanoparticle concentrations in the detection limit were studied. ζ potential measurements and the shift of the surface plasmon band showed a state of saturation with an average number of 55 Lys per gold nanoparticle. Lysozyme–AuNPs interactions induce aggregation of citrate-stabilized AuNPs at low concentrations by neutering the negative charges of citrate anions; from those aggregation data, the magnitude of the interactions has been measured by using Benesi–Hildebrand plots. However, at higher protein concentrations aggregation has been found to decrease. Although the nanocluster morphology remains unchanged in the presence of Lys, slight conformational changes of the protein occur. The influence of the size of the nanoclusters was also investigated for 5, 10, and 20 nm AuNPs, and 10 nm AuNPs was found the most appropriate.

## 1. Introduction

Nanoparticles are characterized by the properties of the metal cluster core, but also by the organic molecules that constitute the monolayer, that is, the capping agents. Gold colloids (AuNPs) are the most stable metal nanoparticles with promising applications because of their electrical, optical, magnetic, and catalytic properties [1]. Besides, gold nanoparticles can be functionalized with a wide variety of structural units by using simple chemical transformations [2,3,4,5]. From the first preparation of gold colloids by Faraday in 1857, research on the use of gold nanoparticles has been an area of broad interest, a fact which is reflected by an exponential growth in the number of publications in the recent years. They are considered largely nontoxic, even though this non-toxicity is strongly dependent on their size and recent publications have reported conflicting data [6]. They are also stable, conductive, catalytically active, and electron dense [7]. Among their many qualities, one stands out greatly: AuNPs possess unique optical properties in terms of extremely high extinction coefficients, distance–dependent color, and outstanding fluorescence quenching ability, popularizing them for biosensor development [8]. Owing to those inherent optical properties (AuNPs possess extinction coefficients which are over 1000 times larger than those of organic dyes) certain chemical interactions lead to aggregation-induced color changes allowing for visual detection and quantification of those interactions [9]. Works dealing with protein detection using AuNPs aggregation-based assays, where both label free and functionalized approaches have been explored, are constantly emerging. The simplicity of these methods should not be ignored; one of their strongest points is the low cost that stems from not requiring sophisticated equipment. In aqueous solution AuNPs can appear with different colors (red, blue, purple) and have a broad absorption peak in the range 500–550 nm, depending on the size of the cluster. This peak is called the surface plasmon resonance (SPR) band [10]. This band depends on several factors and therefore making an analysis is not easy. However, for particles smaller than 30 nm it can be simplified since the phenomenon of resonance when light of adequate wavelength reaches the surface of the nanomaterial produces dipoles and not multipoles, as happens with larger ones. The shape, size, and chemical nature of the nanoparticles are factors that affect the plasmon band, as well as the physical–chemical conditions of the medium in which they are found [11]. This fact makes it possible to analyze the interaction of AuNPs with a ligand in solution if aggregation of the clusters takes place.

Among studies using gold nanoparticles as biosensors, those directed toward the detection of proteins are particularly important. The number of possible proteins that in aqueous media can and have shown affinity for gold nanoparticles, and that are able to induce aggregation and the aforementioned color change is vast and fast-growing. Among them, lysozyme (lys) has proven to be a specially interesting case given its medical relevance: it is virtually absent in the finally excreted urine of healthy subjects, but a urinary excretion of elevated levels of lysozyme (lysozymuria) suggests the existence of tubular disfunction [12]. Increased Lys concentration in urine and serum is associated with leukemia [13], renal diseases [14], and meningitis [15]. Although the present study does not focus on biological systems, instead being limited to lysozyme solutions in water, we believe that a good understanding of the phenomena being reported is essential in order to allow for a future extrapolation of the system to more complex cases, where interferents such as ions or other proteins can be present, as are human body fluids. Only by having a good knowledge of the effects that are directly and exclusively caused by lysozyme on gold nanoparticle systems can the influence of the aforementioned interferents be accounted for and compensated, and to our knowledge no such studies have been carried out as of today. Therefore, we have developed a rigorous study of the interaction of lysozyme with the surface of gold nanoparticles, paying special attention to those factors that can directly impact the performance of the gold colloid systems in potential lysozyme detection systems, such as the stability of the samples, the influence of the nanoparticle size, and the correct way to express gold concentration in the system in order to achieve the most precise and reproducible results. Besides, as a result of our studies, the free energy of binding for the 10 nm AuNPs/Lysozyme interaction in water was determined by surface plasmon resonance (SPR) and a deconvolution method, a procedure that to our knowledge has never been described.

## 2. Materials and Methods

### 2.1. Materials

All chemicals were of Anal. R. Grade. Gold colloid 5 nm (G1402), gold colloid 10 nm (G1527), gold colloid 20 nm (G1652), and lysozyme (L6876) were purchased from Sigma and NaCl from Merck. Solutions were prepared with deionized water, its conductivity being less than 10^−6^ Sm^−1^.

### 2.2. UV-Vis Spectra

The spectra of the AuNPs in the presence and in the absence of lysozyme (Lys) were recorded with a Cary 500 spectrophotometer at 298.2 K from 400 to 700 nm. Nanoparticle concentrations were: [AuNPs] = 8.22 × 10^−10^ M or [AuNPs] = 3.28 × 10^−9^ M (exceptions are indicated in the figure legends). Lysozyme concentrations ranged from 10^−8^ M to 10^−3^ M. Total of 200 µL (or 800 μL, see Results and Discussion) of AuNPs 8.22 × 10^−9^ M was added into a clean quartz cuvette (optical path 10 mm). A timer was started simultaneously as 1800 µL (or 1200 μL) of protein solution of variable concentrations was mixed with the nanosystem solution in the cuvette, which was then reversed several times and then inserted into the cuvette holder in the UV-vis spectrometer. Photograph corresponding to Figure S6 was taken with a Canon 5-50 digital camera.

### 2.3. Zeta Potential Experiments

Zeta potential measurements were carried out at 298.2 K with a Zetasizer Nano ZS Malvern Instruments Ltd (UK). A DTS 1060 polycarbonate capillary cell was used. Figure S1 depicts zeta potential distributions of the 10 nm gold colloids with a negative charge of about −33.1 ± 1.6 mV in water, which is sufficient to keep the particles from interacting with each other and therefore prevent aggregation of the sample, maintaining a stable particle size. Measurements were performed in Lys concentrations range from 10^−8^ M to 3 × 10^−4^ M. [AuNPs] = 8.22 × 10^−10^ M in each case.

### 2.4. Circular Dichroism (CD) Spectra

Electronic CD spectra were recorded in a BioLogic Mos-450 spectropolarimeter. A standard quartz cell of 10 mm path length was used. The spectra were expressed in terms of ellipticity. Scans were taken from 190 nm to 250 nm and for each spectrum 5–10 runs were averaged at a constant temperature of 298.2 K with a 5 min equilibration before each scan. [AuNPs] = 8.22 × 10^−10^ M. [Lys] = 10^−8^ M.

Deconvolution of CD spectra was done by using CDNN software (version 2) with the standard basis set of 33 spectra [16].

### 2.5. Kinetic Measurements

Kinetic runs were carried out in a stopped-flow spectrophotometer from Applied Photophysics. The reaction was monitored by following the changes in absorbance of the surface plasmon band, SPR, at 565 nm, in the presence of [Lys] = 4 × 10^−7^ M and [AuNPs] = 3.28 × 10^−9^ M. A solution of the same AuNPs concentration in water was used as reference. The temperature was maintained at 298.2 ± 0.1 K.

### 2.6. TEM Measurements

For TEM examinations, a single drop (10 μL) of the aqueous solution of the gold nanoparticles was placed on a carbon film-coated copper grid which was then left to dry in air for several hours at room temperature. TEM analysis was carried out in a Philips CM 200 electron microscope working at 200 kV. [AuNPs] = 8.22 × 10^−10^ M; [NaCl] = 0.1 M; [Lys] = 4 × 10^−8^ M.

### 2.7. Deconvolution

Deconvolution of experimental spectra was carried out by fitting to Voigt functions, which best reproduced the SPR of free nanoparticles. Fityk software (0.9 version) was used to carry out the fit [17]. Spectra were fitted to a three-band model, where the free AuNPs band was always found at around 521 nm; the linked AuNPs band appeared at 561 nm for almost all cases except those where the aggregation degree was too small to be significant. A third broad band was fixed at 300 nm in order to account for both residual Au^3+^ absorbance and for the light scattering phenomena linked to the presence of nanoparticles in solution [18].

## 3. Results and Discussion

### 3.1. Stability and Detection Limit Considering the Red Shift of the SPR

**Figure 1:**
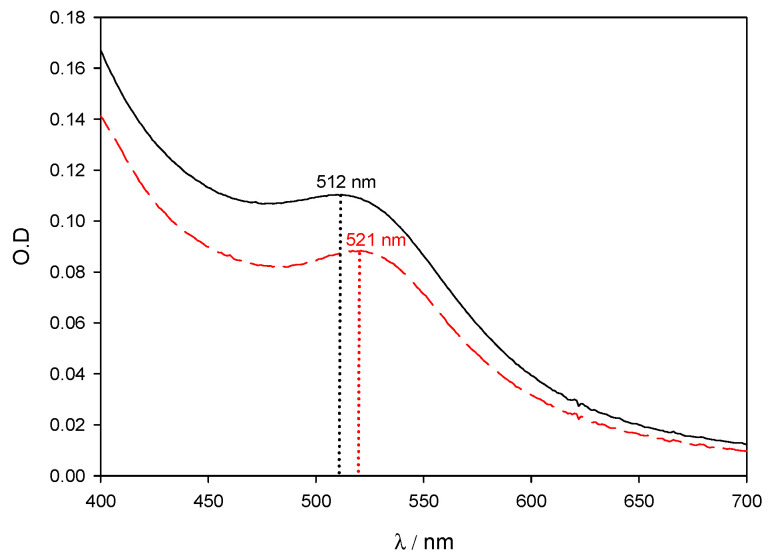
Experimental spectra of AuNPs solutions on the visible region. Commercial gold nanoparticles were used as received. Black solid line corresponds to 4.8 nm AuNPs and gold concentration of 67 μg/mL. Red dashed line corresponds to 5.1 nm AuNPs and gold concentration of 45 μg/mL.

Figure 1 shows the surface plasmon band for two different batches of 5 nm colloidal gold (ref. G1402). The spectra correspond to a mixture of 200 µL of commercial gold with 1800 µL of distilled water. The black solid line corresponds to the batch Lot. SLBB2962V with an average size of 4.8 nm and a gold concentration of 67 µg/mL. The red dashed line corresponds to the batch Lot. 061M6031 with an average size of 5.1 nm and a gold concentration of 45 µg/mL. In order to highlight the influence of gold concentration and nanoparticle size over the SPR band, gold concentrations have not been corrected. It can be clearly seen that a small difference in size (0.3 nm) promotes a change in the maximum of the SPR, (Δλ = 9 nm), and in the optical density (O.D): Δ(O.D) ~ 18%.

**Figure 2:**
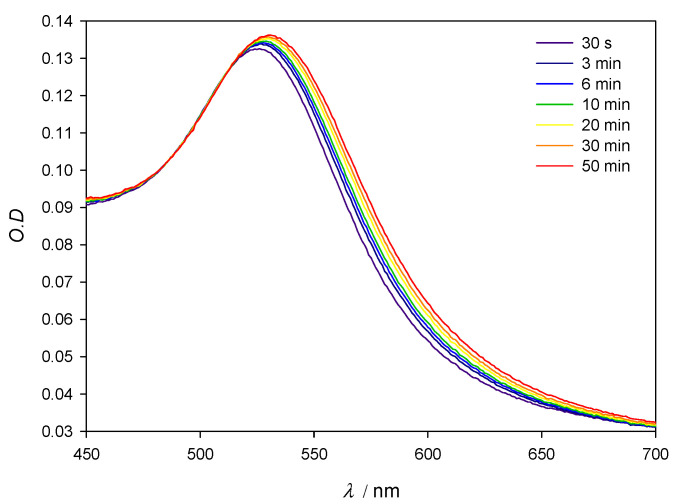
Experimental spectra of 10 nm AuNPs in the presence of Lys 3 × 10^−4^ M. [AuNPs] = 8.22 × 10^−10^ M.

**Figure 3:**
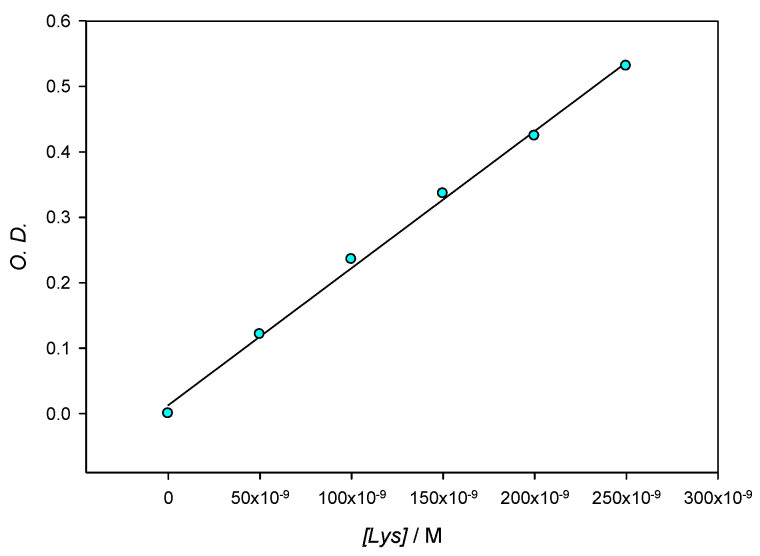
Optical density at different Lys concentrations for a constant ratio [AuNPs]/[Lys] = 0.0164 measured at 565 nm.

Figure 2 shows the SPR stability corresponding to 10 nm AuNPs in the presence of lysozyme 3 × 10^−4^ M. When compared to the SPR of lone AuNPs, λ_max_ shifts to slightly higher values over time when the protein is present in solution; those changes are accompanied by a slight darkening of the red tint of the nanoparticles. Although during the first minutes λ_max_ changes some nanometers, as the optical density does, after the first 10 min. and at least during 1 hour the system’s changes take place at a very slow speed, which allows for the 10 min mark to be taken as a reference in all measurements. For this reason, in further experiments, the values of λ_max_ and O.D. where obtained 10 min after the addition of the protein to the colloidal gold. In order to control the stability of the aggregates, measurements were made at multiple fixed [AuNPs]/[Lys] relationships. Figure 3 reflects compliance with the Lambert–Beer law in the range [AuNPs]/[Lys] = 4.10 × 10^−9^ M/2.50 × 10^−7^ M − 8.22 × 10^−10^ M/5.00 × 10^−8^ M. This implies that while we worked at low concentrations of gold nanoparticles so as to determine the potential lowest detection limits of a lysozyme-sensing system in water, the optical density of the solutions also provides valuable information to this end, and could be explored as an alternate mean for lysozyme quantification.

**Figure 4:**
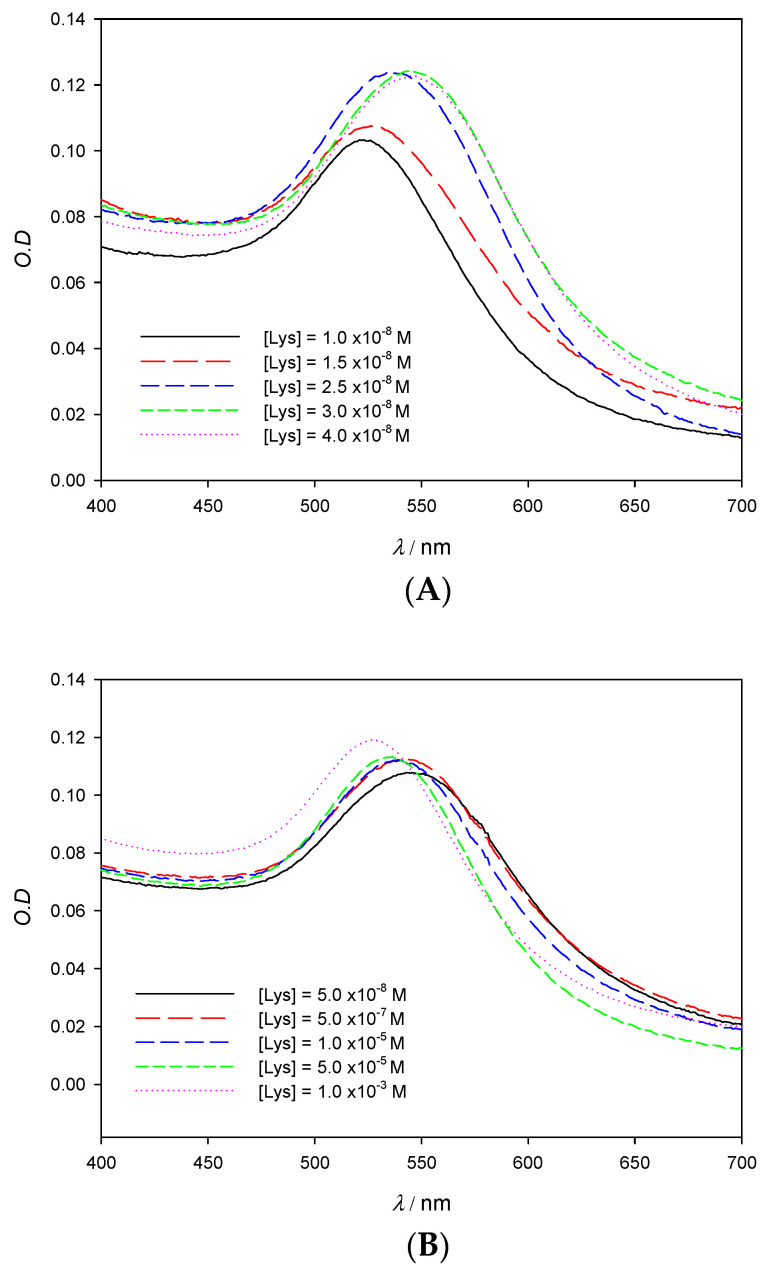
Experimental spectra of 10 nm AuNPs solutions on the visible region showing the red (**A**) and blue (**B**) shift in function of the Lys concentration. [AuNPs] = 8.22 × 10^−10^ M.

**Figure 5:**
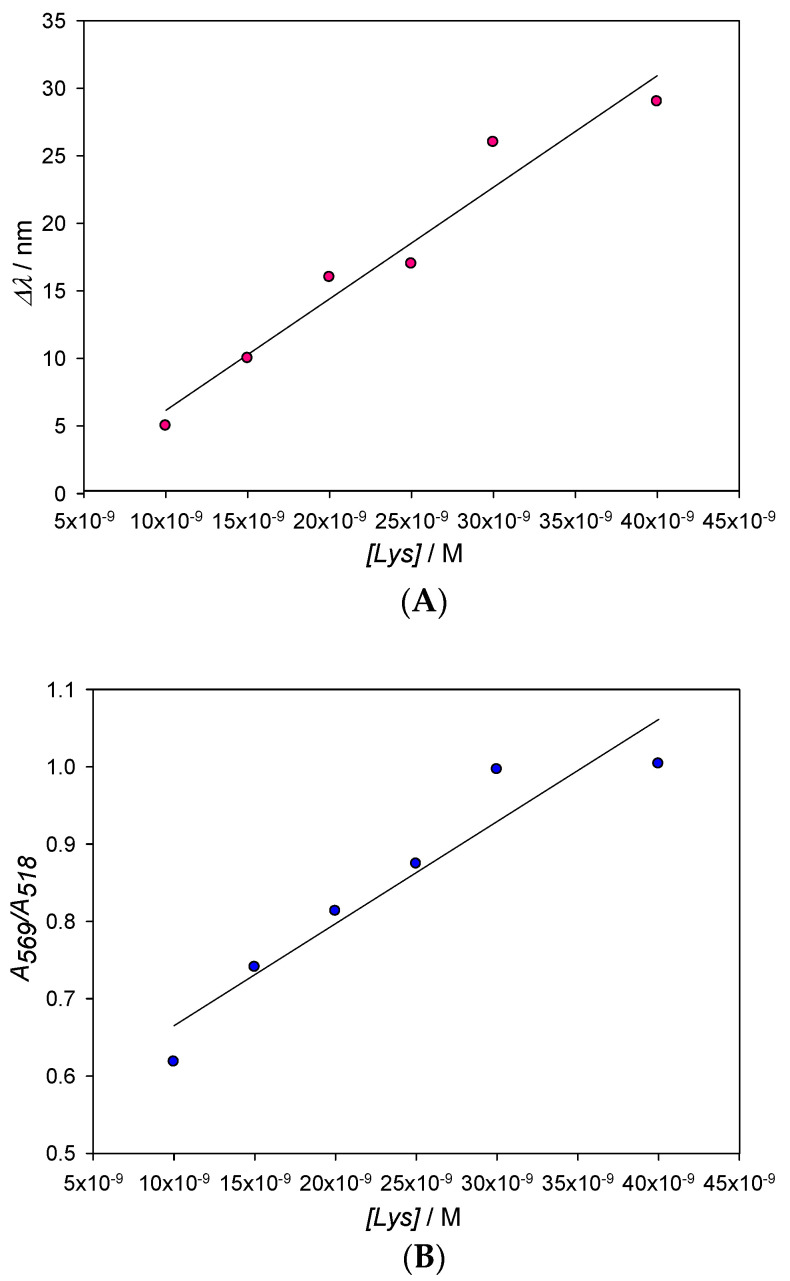
Changes of the maximum of the SPR (**A**) and the A_569_/A_518_ ratio (**B**) with Lys concentration. From Figure 5A, a value of 1.3 × 10^−9^ M for the detection limit (L.D) has been obtained, with L.D. = 3S/D being D the slope of the line and S the standard deviation of the measurements.

The influence of the protein concentration in experimental absorbance λ_max_ measurements is shown in Figure 4A. As can be seen, at low enough concentrations of lysozyme, the SPR experiences a red shift that peaks at [Lys] = 4 × 10^−8^ M; this effect is reflected in Table 1, which shows the values of Δλ at different Lys concentrations when keeping the gold concentration constant. Aggregation phenomena are more marked at low concentrations of the protein, yielding a detection limit in the nanomolar range, around 1.3 × 10^−9^ M. It is also possible to employ the O.D. and consider the degree of aggregation of the nanoclusters in function of the protein concentration (A_569_/A_518_; where A_λ_ stands for absorbance values at λ nm). However, the loss of accuracy can be seen in Figure 5B when compared with Figure 5A. In this study, we have worked with very low gold concentrations, even at the risk of having O.D. ≈ 0.1 in order to achieve high accuracy in the value of λ_max_. Our main goal is to explore the possibilities of using the pronounced λ_max_ variations as an alternative signal instead of A_569_/A_518_ and thus improve the perception of the system changes taking place during the formation of the AuNPs/Lysozyme complex.

The detection limit for lysozyme concentrations in water has been obtained from 3S/D, where S is the standard deviation of the measurements (±0.37) and D the slope of the calibration line (see Figure 5A). Meanwhile, the lower limit of quantification (10 S/D) is 4.3 × 10^−9^ M. Those detection limits, which have been achieved with non-functionalized 10 nm AuNPs, are remarkable when compared to previous studies, even those that use functionalized nanoparticles [19].

### 3.2. Saturation of the System AuNPs/Lys: Structural Characterization

If data of λ_max_ in Table 1 are carefully analyzed, it can be observed that aggregation reaches a state of saturation. Indeed, at higher concentrations of protein, aggregate size begins to decrease; this fact becomes apparent when analyzing the absorbance spectra in Figure 4B, which shows a λ_max_ shift to lower values for lysozyme concentrations higher than 4 × 10^−8^ M. At a given point the nanoparticle surface is saturated and protected all around by a layer of Lys, which prevents aggregation due to steric hindrance and repulsion forces. It is important to note that the concentration of Lys from which λ_max_ starts to decrease matches the absorbance ratio (see Figure S2 in Supporting Information). Considering the AuNPs concentration and the concentration of Lys at which saturation is observed, the average number of protein molecules per nanoparticle has been determined to be 55. Lysozyme has been found to have an estimated size of 3.0 nm × 3.0 nm × 4.5 nm [20]. If the ellipse formed by the longer and one of the shorter diameters, which would have an area of 10.6 nm^2^, is assumed to fully interact with the 10 nm AuNPs surface (314.2 nm^2^), then around 30 lysozyme molecules would fit. If, instead, the shorter diameters are considered, then each lysozyme would take up a circle of 7.07 nm^2^, which would allow around 44 lysozyme molecules to fit. Those theoretical approximations are in accordance with the reported experimental data, since the lysozyme molecules do not need to fully use one of their sides in order to interact with the AuNPs.

**Figure 6:**
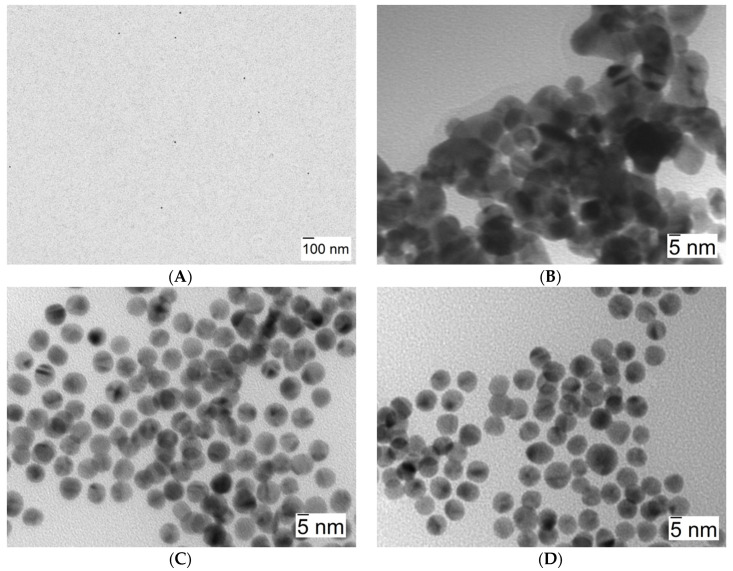
TEM images corresponding to 10 nm AuNPs. (**A**) [AuNPs] = 8.22 × 10^−10^ M, in water (shown at a lesser magnification in order to better convey nanoparticle dispersion). (**B**) [AuNPs] = 8.22 × 10^−10^ M in the presence of [NaCl] = 0.1 M. (**C**,**D**) [AuNPs] = 8.22 × 10^−10^ M in the presence of [Lys] = 4 × 10^−8^ M.

Figure 6 shows three arrays of TEM image photographs that help confirm that the aggregation process takes place as previously stated. It shows images corresponding to solutions in the presence of 10 nm AuNPs (λ_max_ = 518 nm) (A), in the presence of NaCl 0.1 M as the reference aggregation process (λ_max_ = 616 nm) (B), and in the presence of Lys concentration at which the observed shift of λ_max_ is maximum (λ_max_ = 546 nm, see Table 1, [Lys] = 4 × 10^−8^ M) (C). Figure 6C,D clearly shows the aggregation of nanoparticles in the presence of Lys without any apparent changes in nanoclusters morphology.

**Figure 7:**
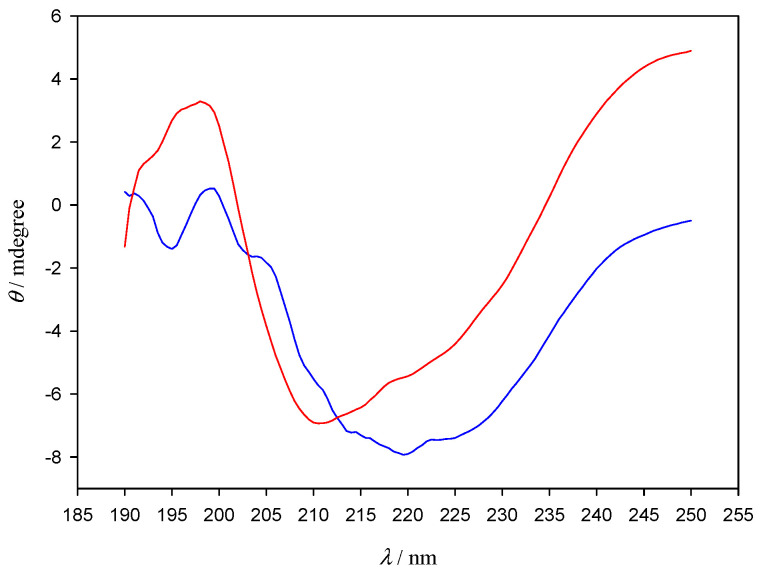
CD spectra of Lys in the absence (red line) and in the presence of 10 nm gold nanoparticles (blue line). [AuNPs] = 8.22 × 10^−10^ M. [Lys] = 10^−6^ M.

In order to further explore the nature of AuNPs–Lys interactions, polarization spectroscopy measurements were carried out (Figure 7). Despite the clear shape difference observed between the lone lysozyme and the lysozyme–AuNPs CD spectra, the deconvolution with CDNN software did not show appreciable changes in the percentages of α-helix, β-turn, and random coil structures present in lone lysozyme in relation to those that form in the presence of AuNPs (see Table S1). Those results are not unexpected due to lysozyme presenting four internal disulphide bonds that confer it a certain degree of stiffness and greatly stabilize its secondary structure, even in the presence of a ligand such as AuNPs. Since there is no appreciable change in the secondary structure of the protein, which strongly depends on those disulphide bonds as well as on intra-molecular hydrogen bonds, we can conclude that the interaction with the AuNPs does not break nor alter either of those links. It is also to note that peptide bonds in proteins are well-known to cause two strong CD bands in the far-UV: one corresponding to π → π* electronic transitions, located around 190 nm, and the other to n → π*, which appears at around 210 nm [21]. Those transitions are clearly visible in the lone lysozyme CD spectra (red line), and show a shift to higher wavelengths, implying lower transition energies, when AuNPs are added (blue line) with a fine structure appearing around 195 nm. We can conclude that even though sulfur atoms have been known to bond strongly with gold [22] the added stability of the disulphide bonds causes AuNPs to preferably interact with the peptidic bond atoms, thus not affecting the secondary structure of the protein. This effect is in agreement with previous reports; for example, 2-(10-mercaptodecyl) malonic acid functionalized 2 nm core gold nanoparticles (anionic AuDA), which form a high affinity complex with Lys, have been found to actually promote re-folding of denatured proteins by shielding them from inter-protein hydrophobic forces; denatured proteins that were treated with AuDA have been shown to present conformations that mimicked those of the native proteins, further proving the point that nanoparticles do not cause significant alterations of the secondary structure of the protein [23]. In any case, the CD results presented in the present study should be considered as an indication, not definitive proof in relation with the secondary structure of the protein.

The stability of different kinds of nanoparticles strongly depends on the valence of the counterions in the solution due to the electrokinetic or ζ potential, this is, the difference between the compact layer potential and the diffuse potential. In this sense, the magnitude of the measured ζ potential is an indication of the repulsive force that prevents nanoparticle aggregation and actually allows the colloidal gold solutions to be stable, and as such can be used to predict the long-term stability of the nanoparticle suspension.

**Scheme 1:**
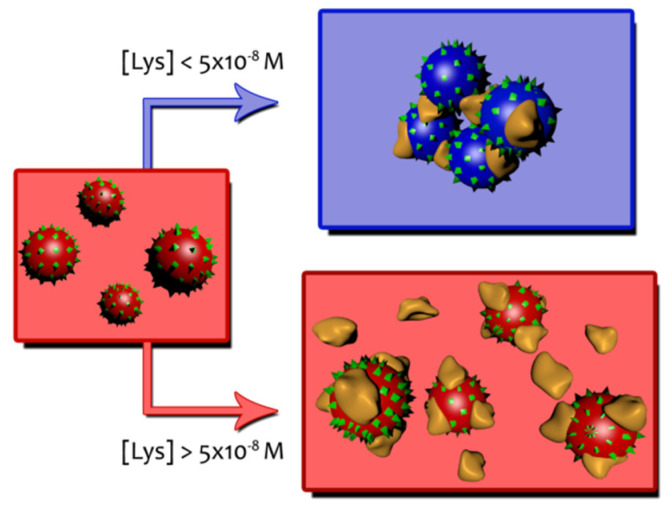
Schematic representation of AuNPs/Lys entities.

Table 2 shows ζ potential values at different Lys concentrations when measured at a constant gold nanoparticles concentration. In the absence of the protein a value of −33 mV (see S1 in supporting information) is sufficient to keep the AuNPs away from each other, thus achieving a stable particle size. ζ potential corresponding to the Lys in the absence of AuNPs indicates that the protein is positively charged at neutral pH (+8.71 mV). As can be seen in Table 2, as the protein concentration increases, the zeta potential becomes less negative, as is to be expected in response to the association of Lys molecules on the gold surface. The partial neutralization of the AuNPs surface decreases the repulsion forces between nanoparticles, facilitating the aggregation. At a concentration around 10^−5^ M a charge reversal occurs, and ζ potential becomes positive. Now AuNPs/Lys entities have a positive average charge and actually begin to repel each other. The situation of maximum aggregation observed from the shifts of the SPR appears at a protein concentration ([Lys] = 4 × 10^−8^–5 × 10^−8^ M) which agrees well with the Lys concentration at which the ζ potential approaches zero. These two behaviors can be described by Scheme 1.

**Figure 8:**
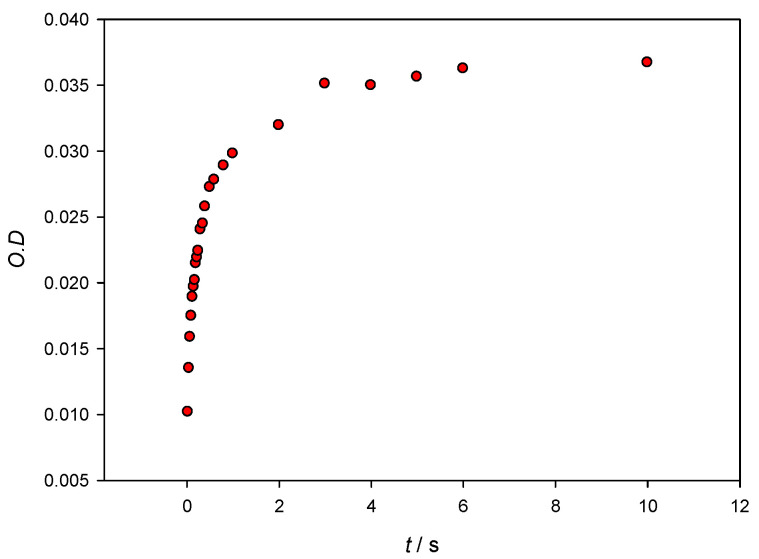
Optical density changes at λ = 565 nm versus time when mixing 10 nm AuNPs with Lys. Concentrations in the stopped flow cuvette are [AuNPs] = 3.28 × 10^−9^ M and [Lys] = 10^−7^ M.

In order to further understand the system, information about the kinetics and the binding free energy of the AuNPs–Lys interaction has been obtained. Figure 8 shows optical density changes versus time at λ = 565 nm for [AuNPs] = 3.28 × 10^−9^ M and [Lys] = 10^−7^ M. Although, for stability reasons, the maxima of the SPR were collected 10 min after mixing the colloidal gold with the protein, it can be seen that, in seconds, it reaches 90% aggregation being only 0.5 seconds necessary to reach an aggregation of 50%. Experimental data cannot be fitted to a monoexponential equation, which evidences that the mechanism of AuNPs–Lys interaction is a complex one.

### 3.3. Influence of the Nanoparticle Size and Gold Concentration. Determination of Binding Free Energy of Lysozyme to Gold Nanoparticles

**Scheme 2:**
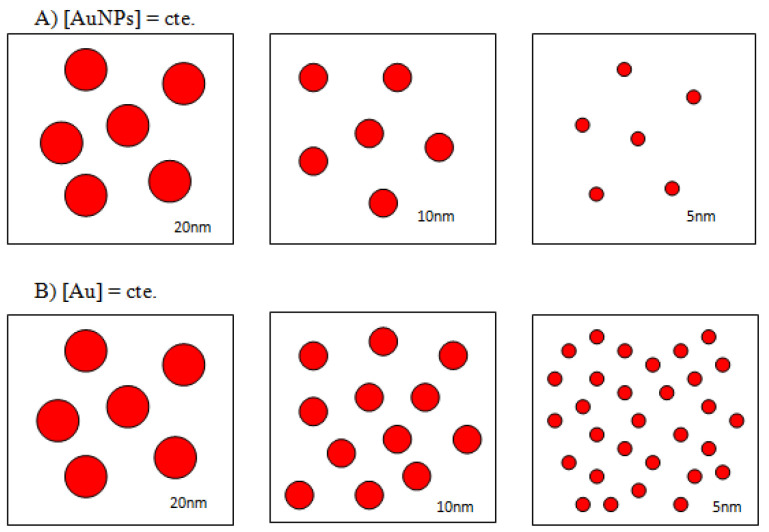
Schematic representation of 5, 10, and 20 nm AuNPs at constant nanoparticles concentration (**A**) and at constant gold concentration (**B**).

A common misconception in gold nanoparticle research papers is that concentration of the colloids is quite frequently expressed in terms of starting gold salt concentrations, so the influence of the particle size is not taken into account. While this approach is correct when working with colloids of a given, monodisperse particle size, in order to study the influence of particle radius into the AuNPs–Lys system working with particle concentrations instead of gold concentrations is a must. As reflected in Scheme 2, varying the particle size at constant gold concentration causes an obvious change in the number of nanosystems in solution. For our purpose of studying the influence of size, it is important to know the number of moles per liter of each nanoparticle size; to this end, the concentration of nanoparticles in each commercial sample was determined as described in the Supporting Information. Our results were practically identical to those of the manufacturer.

Therefore, to analyze the effect of the nanoparticle size in the colorimetric detection process the number of nanoclusters in solution must be kept constant. This situation is reflected in Figure S3A where the SPR of 5, 10, and 20 nm are represented at identical nanoclusters concentrations and in Figure S3B where the SPR corresponds to solutions of identical gold concentrations. As it can be seen, in order to obtain reproducible optical density data, it is essential to increase the concentration of gold well above those used in the experiments described so far. The influence of colloidal gold based on the nanoparticles’ size is a key point in this approach; usually, smaller sized ones are more stable than the larger ones as the latter have higher intensity Van der Waals interactions between them, and these interactions are attractive. Thus, in general, the smaller nanoparticles take longer to aggregate and, once aggregated, may be stable for hours.

The system is stable when working with 20 nm AuNPs and the most dilute solution of Lys (10^−8^ M). However, the observed Δλ was only 1 nm, instead of the 4 nm shift previously reported when 10 nm AuNPs were employed (see Table 1). On the other hand, at high protein concentrations, the system, unlike 10 nm AuNPs, is no longer stable (see Figure S4) showing a precipitation marked by the continuous red shift of λ_max_ and a sudden drop in absorbance.

**Figure 9:**
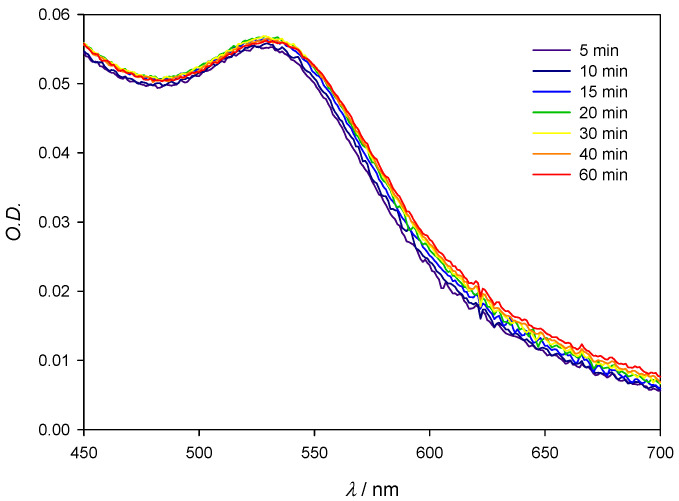
Experimental spectra of 5 nm AuNPs in the presence of Lys. [AuNPs] = 4 × 8.22 × 10^−10^ M; [Lys] = 4 × 10^−4^ M.

As the nanoparticle surface increases, it is necessary to achieve higher protein concentrations in order to aggregate the AuNPs and promote a red shift of the SPR. Thus, a better detection limit is achieved when working with 10 nm AuNPs instead of 20 nm AuNPs. Due to the higher stability of smaller nanoparticles over larger ones, as explained previously, 5 nm AuNPs would be expected to be the most appropriate, but then the ratio Lys/AuNPs should be raised to achieve a reasonable absorbance. The reason is that to compare results at identical concentrations of nanoparticles, 24 µL of 5 nm AuNPs stock solution must be added to 1800 µL of distilled water, compared with 200 µL of 10 nm AuNPs stock solution, and this means having a very low absorbance (see Figure S3A). Figure 9 shows that, instead of 20 nm AuNPs, when working with 5 nm AuNPs and multiplying, for example, the ratio AuNPs/Lys by a factor of 4 relative to 10 nm AuNPs, in order to maintain a constant AuNPs/Lys concentration ratio, the system is stable in the presence of concentrated ([Lys] = 4 × 10^−4^ M) and dilute solutions of protein (data not shown). Even more, with 5 nm AuNPs, a higher detection limit is achieved (Δλ = 7 nm for the lowest concentration of Lys, [Lys] = 10^−8^ M) maintaining the same value of Δλ for [Lys] = 10^−4^ M as that obtained with 10 nm AuNPs in the presence of [Lys] = 3 × 10^−4^ M, that is, Δλ = 11 nm (see Table 1). However, although considering these points 5 nm AuNPs would be the most appropriate to sense the presence of Lys in solution, their wide SPR compared with the better defined SPR of 10 nm AuNPs (see Figure S3B) makes the latter the most appropriate. Results corresponding to 5, 10, and 20 nm are combined into Figure S5.

**Figure 10:**
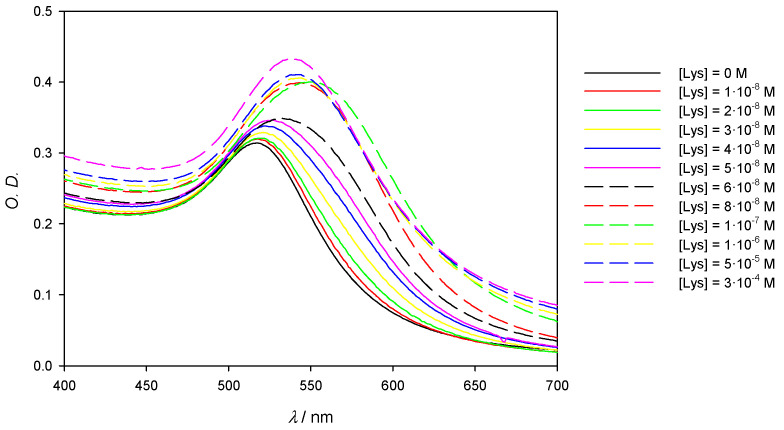
Experimental spectra of 10 nm AuNPs solutions on the visible region showing the red and blue shift in function of the Lys concentration. [AuNPs] = 3.28 × 10^−9^ M. The blue arrow points the shift of the SPB according the Lys concentration increases; the red dashed arrow highlights the reversal of the band at highest concentrations.

With the intention of analyzing the influence of the gold concentration in the AuNPs/Lys system, 10 nm AuNPs have been used at a concentration four times greater than that employed throughout this study. Figure 10 and Table 3 show the obtained results. Note that the red and the blue shift are once again observed, depending on the Lys/AuNPs ratio. As the detection limit worsens (Δλ = 1 nm for [Lys] = 10^−8^ M), larger values of Δλ are achieved at high Lys concentrations, as in this case the saturation of the AuNPs surface is reached at higher protein concentrations. Naturally, as shown in a comparative photograph, the color changes are more intense and defined than when samples of more dilute gold are used (see Figure S6).

**Figure 11:**
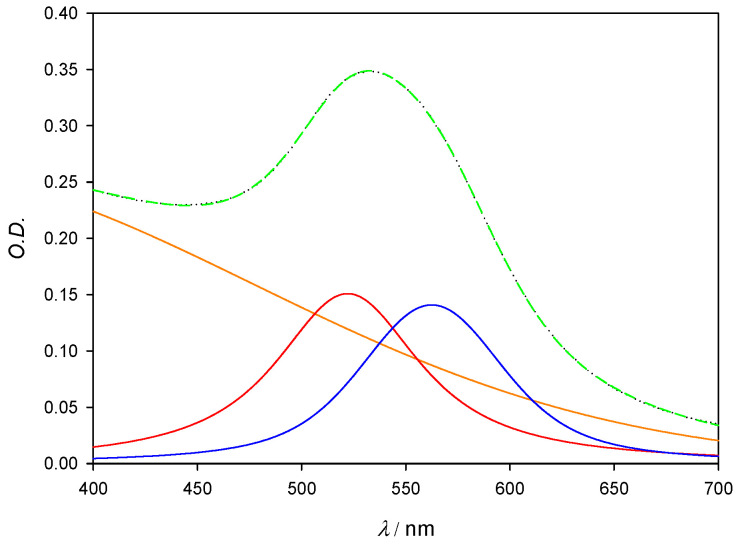
Deconvolution plot for [AuNPs] = 6 × 10^−8^ M. Orange line corresponds to residual Au^3+^ salts; red line corresponds to the SPR of free gold nanoparticles and peaks at 522 nm; blue line shows the SPR that arises from the formation of the gold aggregates. Black (dotted) line corresponds to the experimental spectra and green (dashed) line corresponds to the sum of all deconvoluted bands.

From the spectra shown in Figure 10, deconvolution procedures were carried out in order to obtain a better view of both SPRs: the one corresponding to non-interacting, free AuNPs, which show a band whose peak is already known to be located at around 521 nm; and the one from aggregated nanoparticles, whose width makes it hard to determine the exact location of its absorbance maxima. The deconvoluted peak sum shows a near perfect agreement with experimental data when the fit is done with a three peak model, as shown in Figure 11; the third peak is fixed at 300 nm and accounts for both residual Au^3+^ salts (which show a broad band centered around that wavelength) and for the dispersion effects that happen in the near-UV and are inherent to gold nanoparticle solutions [18]. It is also important to note that the λ_max_ of the aggregation band may shift with increasing concentrations of lysozyme, as the size distribution of aggregates becomes more disperse and the band widens. However, this effect was not observed in our system, as reflected in Table 4, and the aggregation band maxima remained at 561 ± 1 nm for all measurements except [Lys] = 2 × 10^−8^ M, where the aggregation degree is still very low.

The evolution, both in area and in maxima intensity, of the two deconvoluted peaks is directly linked to the fraction of AuNPs that are bound to lysozyme for each concentration, although the direction in which this change takes place shifts in accordance to the aggregation–disaggregation effects mentioned earlier. When aggregation phenomena are prevalent, the intensity of the aggregated deconvolution band at 561 nm grows with increasing [Lys]. Past the turning point of 1 × 10^−7^ M, increasing [Lys] causes the aggregation band to shrink, because the excess lysozyme has the effect of preventing aggregation, as previously discussed.

Since the absorbance of the aggregated AuNPs band (and by extent, that of the free AuNPs band) is directly linked to the concentration of aggregated AuNPs, those absorbance data offer a reliable way of determining the binding constant, and by extent the binding free energy, ΔG^0^, of the AuNPs/Lys complex by using a simplified reaction scheme:

(1) $$AuNPs+Lys\;\overset{K_{b}}{↔}\;AuNPs/Lys$$

where *K_b_* is the binding constant of our system.

The stable position of the absorbance peak allows for the binding constant to be determined by using the Benesi–Hildebrand equation [24], which uses absorbance measurements at a fixed wavelength to monitor the formation of a colored complex during a chemical reaction:

(2) $$\frac{{\left[AuNPs\right]}_{0}}{Abs}=\frac{1}{K_{b}\varepsilon \;{\left[Lys\right]}_{0}}\frac{1}{\varepsilon }$$

where *K_b_* is the formation constant of the complex, and *ε* its molar extinction coefficient. For this model, it is assumed that the absorbance band of the complex does not shift during the formation process.

**Figure 12:**
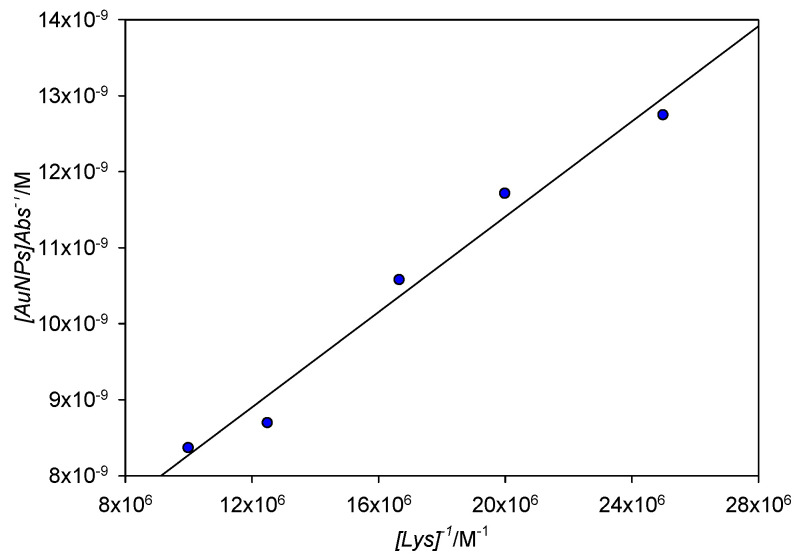
Benesi–Hildebrand plot for the absorbance at 561 nm for [Lys] = 4 × 10^−8^ M^−1^ × 10^−7^ M.

Figure 12 shows a Benesi–Hildebrand plot of the absorbance measured at 561 nm for [Lys] = 4 × 10^−8^ M to 1 × 10^−7^ M; lower concentration points showed slight deviations of linearity, probably due to the low concentration of aggregated nanoparticles in solution. From the fit, the value obtained for K_b_ was 1.64 × 10^7^ M^−1^, which corresponds to a free energy of binding ΔG^0^ = −41.2 kJ mol^−1^. Extinction coefficient (ε) of the aggregated AuNPs was also calculated, finding a value of 1.95 × 10^8^ M^−1^cm^−1^. When the extinction coefficient of 10 nm free AuNPs was estimated through theoretical means [25], a value of 1.03 × 10^8^ M^−1^cm^−1^ was found, remarkably close to that of our experimental measurements.

**Figure 13:**
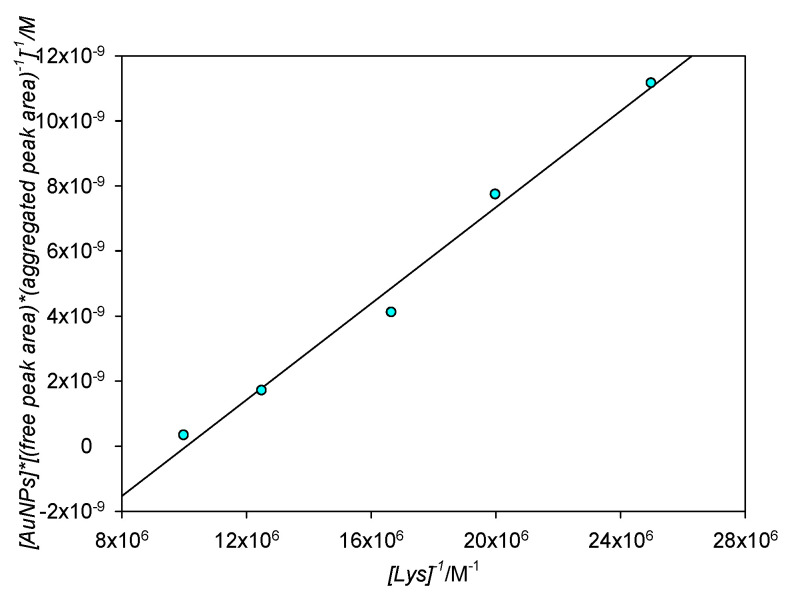
Benesi–Hildebrand plot for aggregated/free area quotients for [Lys] = 4 × 10^−8^ M^−1^ × 10^−7^ M.

In order to corroborate those results, the Benesi–Hildebrand equation was also applied to the area quotient between the aggregated and the free peaks. Since this fitting model was developed in order to be used in cases where the complex formation is directly proportional to the receptor concentration (in our case, [Lys]) by virtue of the Lambert–Beer law, the aggregated/free area ratio would also need to be directly proportional to [Lys] for the model to be valid. It is known, from the previous fit, that the aggregated peak intensity is directly proportional to [Lys]; knowing that the formation of the aggregate is directly linked to the disappearing of the free SPR, and supposing that no band widening takes place and both the aggregated and free peaks remain roughly the same shape, then it follows that the area of both peaks should be directly proportional to one another and by extension to [Lys], albeit the proportionality constant will not correspond to ε since this constant is linked to absorbance and not area. If the previous statements are true, then the Benesi–Hildebrand plot for the aggregated/free area quotient should also be a straight line; experimental results are shown in Figure 13. As can be seen, the fit of the area quotient to the Benesi–Hildebrand equation is good (R^2^ = 0.988). The K_b_ for this fit is 1.20 × 10^7^ M^−1^, corresponding to ΔG^0^ = −40.4 kJ mol^−1^ and so in a really good agreement to that obtained by classical Benesi–Hildebrand representations.

## 4. Conclusions

A thorough study of AuNPs–Lys interaction in aqueous solution has been carried out. Aggregation occurs in seconds, but it has been found that at high protein concentrations the aggregation degree of the AuNPs markedly decreases, a fact confirmed by the blue shift of λ_max_, the observed color changes of AuNPs/Lysozyme solutions from purple to red, and by the changes of ζ-potential, going from a negative to a positive value as [Lys] increases. The state of saturation has been found to imply an average number of 55 Lys per gold nanoparticle, in good accordance with both the protein and the AuNPs size. Although the nanocluster morphology remains unchanged in the presence of Lys, conformational changes of the protein occur, but the secondary structure of the protein remains unchanged, which indicates that no lysozyme disulphide bonds break from the interaction with AuNPs. From experimental absorbance data, by using Benesi–Hildebrand plots of both A_561_ and deconvoluted free AuNPs/aggregated AuNPs absorbance area quotients, the free energy of binding (ΔG^0^) of lysozyme to 10 nm AuNPs has been found to be around −40.8 kJ mol^−1^ with a good agreement between the two fits; to our knowledge, this work is the first instance of deconvolution parameters being used in order to determine the binding constant of lysozyme to gold nanoparticles. Studies of the influence of AuNPs size on the interaction with lysozyme confirm the need of using nanoparticle concentrations instead of gold concentrations when approaching the study of a new nanosystem, and also show that 10 nm AuNPs are the most appropriate for those studies. Working with high gold concentrations does not improve the results achieved, although better defined changes in color are accomplished.

## Figures and Tables

**Table 1:** Values of Δλ/nm at different protein concentrations. [AuNPs(10 nm)] = 8.22 × 10^−10^ M.

[Lys]/M	Δλ/nm	[Lys]/M	Δλ/nm
1.0 × 10^−8^	4	5.0 × 10^−7^	24
1.5 × 10^−8^	9	1.0 × 10^−6^	22
2.0 × 10^−8^	15	1.0 × 10^−5^	21
2.5 × 10^−8^	16	5.0 × 10^−5^	15
3.0 × 10^−8^	23	1.0 × 10^−4^	13
4.0 × 10^−8^	28	3.0 × 10^−4^	11
5.0 × 10^−8^	27	1.0 × 10^−3^	8
1.0 × 10^−7^	23	-	-

**Table 2:** Values of ζ potential/mV at different protein concentrations. [AuNPs (10 nm)] = 8.22 × 10^−10^ M. ζ Potential = +8.71 mV for Lys in the absence of AuNPs.

[Lys]/M	ζ Potential/mV
0	−33.0
1.0 × 10^−8^	−30.1
2.0 × 10^−8^	−29.9
3.0 × 10^−8^	−22.6
4.0 × 10^−8^	−13.8
5.0 × 10^−8^	−10.5
1.0 × 10^−6^	9.4
5.0 × 10^−5^	15.4
3.0 × 10^−4^	10.1

**Table 3:** Values of Δ λ/nm at different protein concentrations. [AuNPs (10 nm)] = 3.28 × 10^−9^ M.

[Lys]/M	Δλ/nm	[Lys]/M	Δλ/nm
1.0 × 10^−8^	1	8.0 × 10^−8^	27
2.0 × 10^−8^	2	1.0 × 10^−7^	32
3.0 × 10^−8^	4	1.0 × 10^−6^	27
4.0 × 10^−8^	5	5.0 × 10^−5^	27
5.0 × 10^−8^	10	3.0 × 10^−4^	21
6.0 × 10^−8^	16	-	-

**Table 4:** Values of λ_max_/nm for both free and aggregated AuNPs at different protein concentrations.

[Lys]/M	λ_max_ (Free AuNPs)/nm	λ_max_ (Aggregated AuNPs)/nm
1.0 × 10^−8^	521	0
2.0 × 10^−8^	521	559
3.0 × 10^−8^	521	561
4.0 × 10^−8^	521	562
5.0 × 10^−8^	521	561
6.0 × 10^−8^	521	562
8.0 × 10^−8^	521	562
1.0 × 10^−7^	521	561

## Supplementary Materials

The following are available online at https://www.mdpi.com/article/10.3390/nano11082139/s1, Table S1: Deconvoluted structures of native lysozyme and the AuNPs-lysozyme complex, Figure S1: ζ-Potential of 10 nm AuNPs, Figure S2: (A) Changes of the maximum of the SPB (λ_max_). (B) Changes of the ratio A_569_/A_518_ at each protein concentration of Table 1. [AuNPs] = 8.22 × 10^−10^ M, Figure S3: (A) SPB of 5, 10 and 20 nm AuNPs at the same nanoparticle concentration. [AuNPs] = 8.22 × 10^−10^ M. (B) SPB of 5, 10 and 20 nm AuNPs at the same gold concentration. [Au] = 50 μ, Figure S4: Stability study of 20 nm AuNPs in the presence of [Lys] = 10^−4^ M. [AuNPs] = 8.22 × 10^−10^ M, Figure S5: (A) Stability study of 5 nm AuNPs in the presence of [Lys] = 4 × 10^−4^ M. [AuNPs] = 4 × 8.22 × 10^−10^ M; (B) Stability study of 10 nm AuNPs in the presence of [Lys] = 3 × 10^−4^ M. [AuNPs] = 8.22 × 10^−10^ M. (C) Stability study of 20 nm AuNPs in the presence of [LYS] = 10^−4^ M. [AuNPs] = 8.22 × 10^−10^ M, Figure S6: A comparative analysis corresponding to the first nine Lys concentrations of Table 3 in the presence of [AuNPs] = 8.22 × 10^−10^ M and [AuNPs] = 3.28 × 10^−9^ M.
