# Supplementary material for: Lysozyme–AuNPs Interactions: Determination of Binding Free Energy
Source: Nanomaterials (Basel). 2021 Aug 22;11(8):2139. doi: 10.3390/nano11082139 (PMC8400155; doi:10.3390/nano11082139)
Supplement: Supplementary file 1 [file nanomaterials-11-02139-s001.zip › nanomaterials-1339913-supplementary.pdf]

nanomaterials MDPI

Supplementary Materials

Lysozyme–AuNPs Interactions: Determination of Binding Free Energy

Axel Gomes [1], Jose M. Carnerero [1], Aila Jimenez-Ruiz [1], Elia Grueso [1], Rosa M. Giráldez-Pérez [2] and Rafael Prado-Gotor [1,*]

[1] Department of Physical Chemistry, Faculty of Chemistry, University of Seville, Seville, 41012 Spain; axelgomes314@hotmail.fr (A.G.); jcarnerero2@us.es (J.M.C.); ajimenez28@us.es (A.J.-R.); elia@us.es (E.G.)
[2] Department of Cellular Biology, Physiology and Immunology, Faculty of Science, University of Córdoba, Cordoba, 14014, Spain; rgiraldez@uco.es (R.M.G.-P.)
* Correspondence: pradogotor@us.es

Determination of the concentration of AuNPs

Technical commercial data sheet provided by Sigma-Aldrich indicates the nanoparticle´s core diameter. The mean number of Au atoms in a particle, n, can be calculated by using the following relationship[1]:

$$n = \frac{0.5\,\pi\, N_A\, d_m^3}{3\, V_m} \tag{1}$$

In the above equation, which assumes a spherical particle shape, N_A is the Avogadro number, d_m is the diameter of the nanoparticle expressed in cm, and V_m is the molar volume of bulk gold (10.215 cm^3).[2] For example, for 10 nm gold nanoparticles the mean number of Au atoms in a particle corresponds to n = 30867. Once the concentration of Au atoms in the nanoparticles solution is known (as supplied by Sigma-Aldrich) it is possible to obtain the average concentration of nanoparticles. Results following this method are almost similar to those obtained by Sigma-Aldrich.

Table S1. Deconvoluted structures of native lysozyme and the AuNPs–lysozyme complex.

	**Lysozyme**	**Lysozyme-AuNPs**
**α-Helix**	10,4 %	9,8 %
**Antiparallel β sheet**	31,4 %	31,5 %
**Parallel β sheet**	4,0 %	4,0 %
**Beta-Turn**	20,6 %	18,5 %
**Random Coil**	36,2 %	36,0 %
**Total Sum**	102,7 %	99,8 %

[1] S. L. Cumberland,G. F. Strouse, *Langmuir* **2002**, *18*, 269–276.
[2] A. Henglein, M. Giersig, *J. Phys. Chem. B* **1999**, *103*, 9533–9539

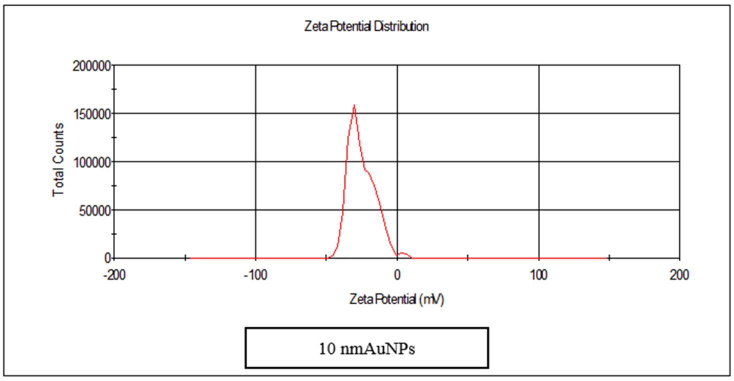

Figure S1. ζ-Potential of 10 nm AuNPs.

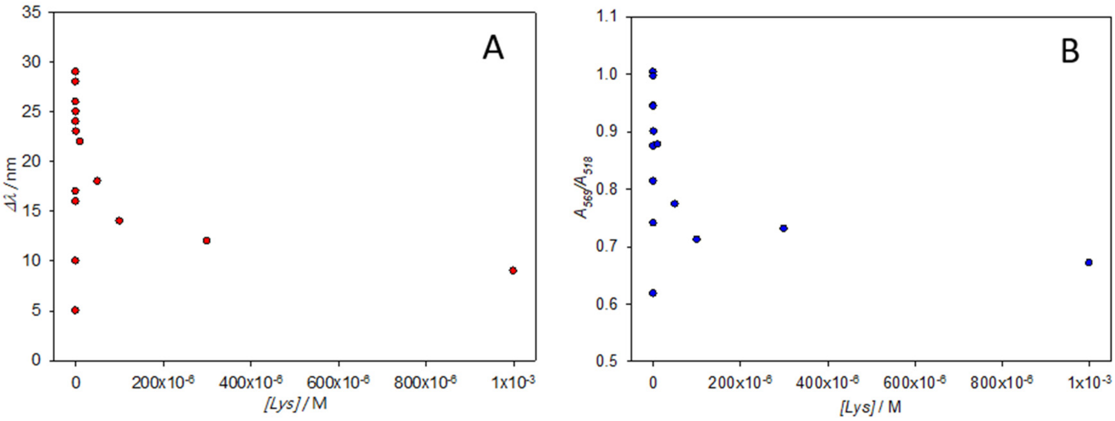

Figure S2. (**A**) Changes of the maximum of the SPB (λ_{max}). (**B**) Changes of the ratio A_{569}/A_{518} at each protein concentration of Table 1. [AuNps] = 8.22×10^{-10} M.

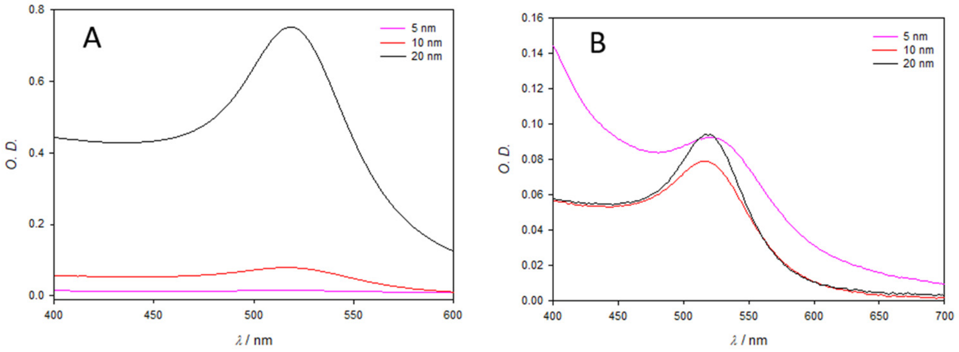

Figure S3. (**A**) SPB of 5, 10, and 20 nm AuNPs at the same nanoparticle concentration. [AuNPs] = 8.22×10^{-10} M. (**B**) SPB of 5, 10, and 20 nm AuNPs at the same gold concentration. [Au] = 50 μg/mL.

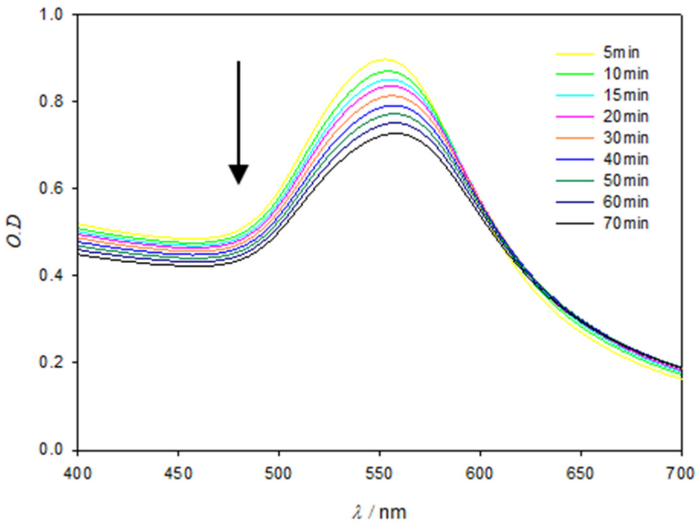

Figure S4. Stability study of 20 nm AuNPs in the presence of [Lys] = 10^{-4} M. [AuNPs] = 8.22×10^{-10} M.

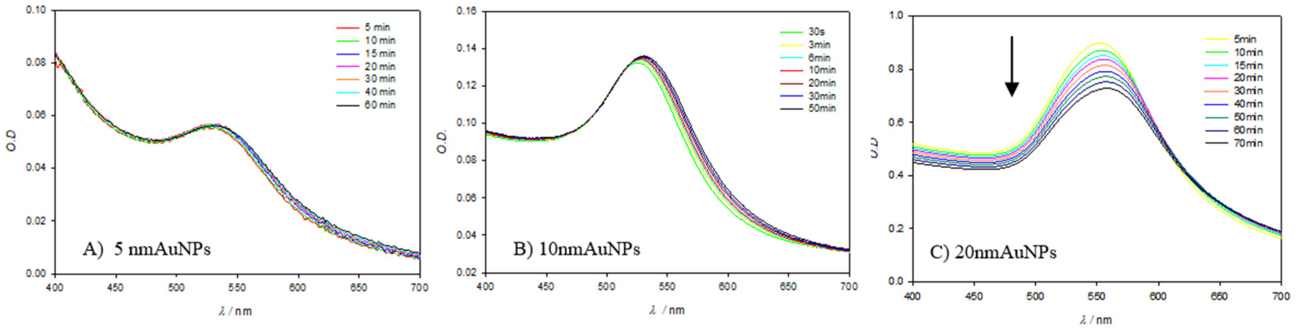

Figure S5. (**A**) Stability study of 5 nm AuNPs in the presence of [Lys] = 4×10^{-4} M. [AuNPs] = $4 \times 8.22 \times 10^{-10}$ M. (**B**) Stability study of 10 nm AuNPs in the presence of [Lys] = 3×10^{-4} M. [AuNPs] = 8.22×10^{-10} M. (**C**)Stability study of 20 nm AuNPs in the presence of [LYS] = 10^{-4} M. [AuNPs] = 8.22×10^{-10} M.

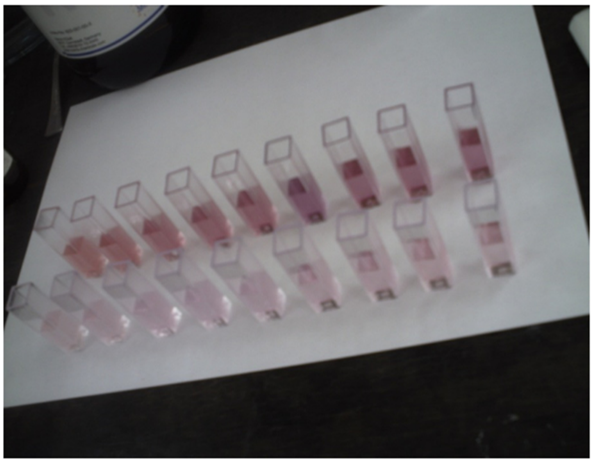

Figure S6. A comparative analysis corresponding to the first nine Lys concentrations of Table 3 in the presence of [AuNPs] = 8.22×10^{-10} M and [AuNPs] = 3.28×10^{-9} M.
